# A cross-sectional seroepidemiology study of EV-D68 in China

**DOI:** 10.1038/s41426-018-0103-4

**Published:** 2018-06-06

**Authors:** Shiyang Sun, Fan Gao, Yalin Hu, Lianlian Bian, Xing Wu, Yao Su, Ruixiao Du, Ying Fu, Fengcai Zhu, Qunying Mao, Zhenglun Liang

**Affiliations:** 1 National Institute for Food and Drug Control, Beijing, China; 2 Hualan Biological Engineering Inc, Xinxiang, China; 3 Jiangsu Provincial Center for Disease Control and Prevention, Nanjing, China

## Abstract

Enterovirus 68 (EV-D68) is associated with respiratory diseases, such as acute upper respiratory tract infections (URTIs), lower respiratory tract infections (LRTIs), pneumonia, neurological diseases, and acute flaccid myelitis (AFM). In recent years, there have been global outbreaks of EV-D68 epidemics. However, there is no effective vaccine against EV-D68, and the understanding of the seroprevalence characteristics of EV-D68 is limited. To evaluate the epidemiological features of this emerging infection in mainland China, serum samples from 20 pairs of pregnant women and their neonates, 405 infants and children (ages 1 month–15 years), and 50 adults were collected to measure EV-D68 neutralizing antibodies (NtAbs). The results showed that the geometric mean titers (GMTs) of pregnant women and their neonates were 168 (95%CI: 93.6–301.7) and 162.3 (95%CI: 89.9–293.1), respectively. The seroprevalence rate of EV-D68 antibodies was negatively correlated with age in 1-month-old to 12-month-old infants (84% for 1-month-old infants vs 10% for 1-year-old infants), whereas it was positively correlated with age for 1-year-old to 15-year-old children (10% for 1-year-old children vs 92% for 15-year-old children). This study evaluated maternal antibodies against EV-D68 in neonates. Our results showed that if mothers had high levels of anti-EV-D68 NtAbs, the NtAbs titers in their neonates were also high. The GMTs and seroprevalence rates of each age group indicated that EV-D68 infection was very common in China. Periodical EV-D68 seroprevalence surveys and vaccination campaigns should be the top priority for preventing EV-D68 infection.

## Introduction

Human enteroviruses (HEVs) are classified into 12 species, including enteroviruses A through J and rhinoviruses A through C^1^. EV-D68 belongs to enterovirus D. As a non-enveloped, positive-sense, single-stranded RNA virus, EV-D68 has a genome that contains a single open reading frame coding for a poly-protein (P1), the precursor of four viral capsid proteins, VP1, VP2, VP3, and VP4, and seven non-structural proteins, 2A, 2B, 2C, 3A, 3B, 3C, and 3D. VP1 and VP3 are the major antigenic epitopes^2, 3^.

EV-D68 was first isolated in California from children with pneumonia and bronchiolitis in 1962^4^. Since then, EV-D68 infections have been identified only sporadically around the world. For example, there were only 26 cases of documented EV-D68 respiratory disease in the United States from 1970 to 2005^5^. However, the upsurge of EV-D68 cases in the past few years showed clusters of infections in Europe, the Americas, Asia, Oceania and Africa^6–8^. In particular, more than 1000 cases, including 14 deaths, were reported during the epidemic of EV-D68 infection in 2014 in the United States^9^, resulting in strong public attention toward this virus. During August 2006–April 2010 in Beijing, China, coxsackievirus A21 and enterovirus 68 were detected in enterovirus-positive adults with acute respiratory tract infections, and the EV-D68 positive rate was 10% (13/130)^10^. EV-D68 was also reported in other areas of China^11–14^. More cases were reported in different geographical locations in the following years^11, 15^. EV-D68 infection was predominantly found in pediatric patients and caused a wide range of symptoms: fever, runny nose, sneezing, cough, skin rash, and body/muscle aches. It could lead to severe acute upper respiratory tract infections (URTIs), lower respiratory tract infections (LRTIs), pneumonia, neurological illness, acute flaccid myelitis (AFM), or even death^6, 15–17^. There is no effective vaccine, medicine or treatment that can prevent this virus from spreading.

In humans, humoral immunity with neutralizing antibodies is crucial for protection against EV-D68 infection^18^. Unfortunately, immunogenicity in maternal sera and the pattern of immune responses against EV-D68 have not been well studied in mainland China. Further study is necessary to understand the distribution of immunogenicity against EV-D68 infection. To investigate the seroprevalence of EV-D68 infection in Jiangsu province, China, we conducted a cross-sectional study. Trans-placental serum from prenatal women and serum from their neonates were collected and analyzed to identify the age-specific seroprevalence rate of EV-D68 infections in infants/children (ages 1 month to 15 years). All serum samples were collected in August 2010 in Donghai County in Jiangsu Province, China^19^. In addition, the seroprevalence of EV-D68 infection in adults was investigated using adult serum provided by Hualan Biological (Fengqiu) single-mining Plasma Co., Ltd, in 2015.

## Results

### EV-D68 Virus preparation

**Fig. 1:**
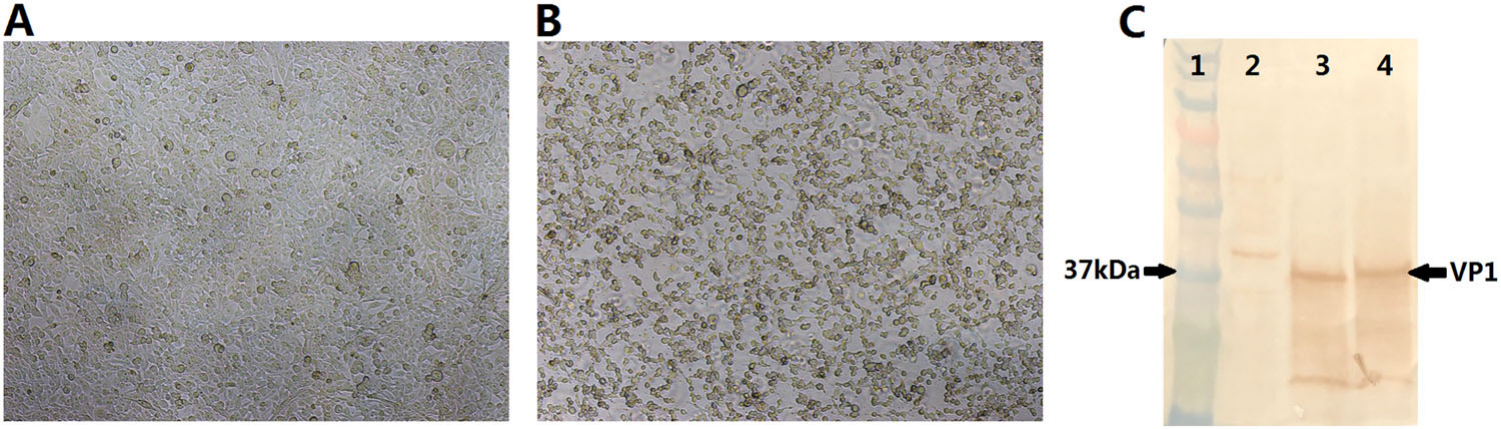
Cytopathic effects observed in RD cells (×10). **a** Control. **b** Cells infected with the synthetic virus. **c** Western blotting using anti-EV-D68 VP1 antibodies. Lane1: marker; lane2: cell control; lane3: Fermon virus; lane4: synthetic virus

Reverse genetics was used to produce the EV-D68 virus. Two plasmids were transfected into 293T cells with Lipo 2000. After 3–4 days of incubation, the cells were frozen and thawed. The supernatant was harvested and used to infect the RD cells. The infected RD cells were cultured for five to seven days and periodically examined for viral cytopathogenic effect (CPE) (Fig. 1a, b). The viruses were harvested from the cultured cells, and the tissue culture infective dose of 50% (TCID_50_) was determined. The virus titers of the synthetic virus and the Fermon strain were 10^8^ and 10^7.5^ TCID_50_/ml, respectively. Western blotting showed that the prepared virus contained a special band of EV-D68 VP1 with the Fermon strain as the positive control (Fig. 1c), indicating that the synthetic virus produced by the reverse genetics method could cause CPE in RD cells with high titers and could be used as a challenge virus in the EV-D68 NtAb assay.

### NtAbs assay for EV-D68 using the synthetic virus strain or the Fermon strain

To confirm the specificity of the EV-D68 synthetic virus strain, the NtAbs of the anti-serum of other enteroviruses, such as hepatitis A, enterovirus 71, coxsackievirus A16, coxsackievirus A6, coxsackievirus A10, coxsackievirus B3, and coxsackievirus B5 (stored in our laboratory), were tested with the synthetic virus. The results showed that the synthetic virus could not be neutralized by any anti-serum for other enteroviruses (data not shown). This result demonstrated that the synthetic virus strain had good specificity.

**Fig. 2:**
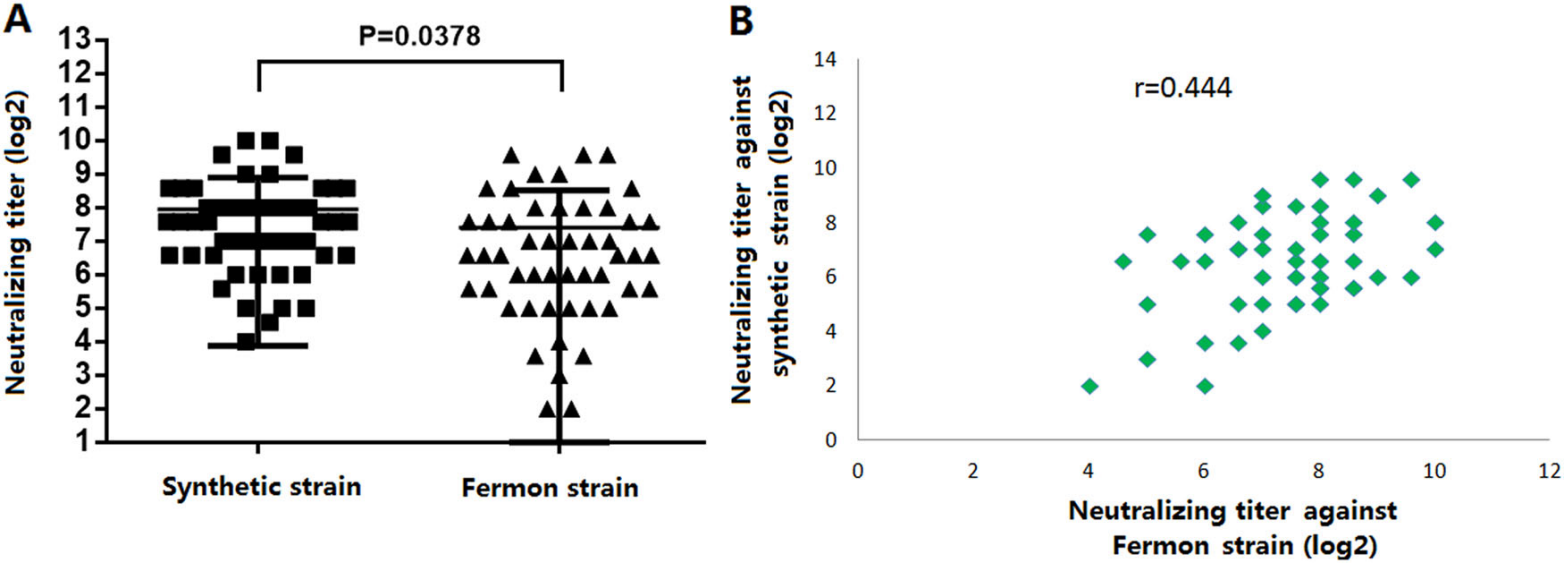
Comparison of the synthetic virus and the Fermon strain in NtAbs using 50 adult serum samples. **a** Comparison of the NtAbs of anti-EV-D68 against the synthetic virus and Fermon (*p* = 0.0378). **b** The correlation of NtAbs against EV-D68 between the synthetic virus and Fermon (*r* = 0.444)

The difference between NtAbs assays using the synthetic virus strain and the Fermon strain was investigated. The EV-D68 NtAbs for 50 healthy adult sera were measured using both the synthetic virus strain and the Fermon strain. The results showed that a 100% seroprevalence rate (50/50) was found for both challenge viruses. The NtAbs GMTs were 166.3 (95% CI 126.8–218.0) and 88.6 (95% CI 61.9–128.3) against the synthetic virus strain and the Fermon strain, respectively (Fig. 2a). The synthetic virus strain from China showed higher NtAbs GMTs than the Fermon strain from America (*p* = 0.0378), which was in accordance with the epidemiology. The statistical analysis showed that there was good correlation between NtAb titers against the synthetic virus strain and against the Fermon strain (*r* = 0.444) (Fig. 2b). Therefore, the synthetic virus was chosen as the challenge strain in the subsequent research.

### Seroprevalence rate and GMTs of anti-EV-D68 in blood samples from prenatal women and their neonates

To determine the maternal antibodies, serum samples from 20 pairs of women and their neonates were collected. The EV-D68 NtAbs were tested using the synthetic viruses (Table 1). The results showed that the seroprevalence rates of EV-D68 NtAbs were 100% (20/20) in both prenatal women and their neonates. The GMTs of EV-D68 NtAbs were 168.0 and 162.3 in prenatal women and their neonates, respectively. The GMT difference between prenatal women and their neonates was not statistically significant (*p* = 0.817).

**Table1:** Seroprevalence rates and GMTs of NtAbs from the blood samples of 20 pairs of prenatal women and their neonates

Subjects	Seroprevalence rates	GMTs	95% CI
Prenatal women	100% (20/20)	168.0	93.6–301.7
Neonates	100% (20/20)	162.3	89.9–293.1

To further analyze the relationship of antibody titers between prenatal women and their neonates, we classified NtAbs titers into four groups: negative (<8), low (8–64), moderate (96–512) and high (>512). The percentages of infants in each group are shown in Table 2. The results showed that the NtAbs titers of eight pairs (20%) of mothers and their neonates were in the range of 1:8 to 1:64, the NtAbs titers of 20 pairs (50%) were in the range of 1:96 to 1:512, and the NtAbs titers of four pairs (10%) were above 1:512. There was a positive correlation between EV-D68 NtAbs in neonates and those in their mothers (*r* = 0.76, *p* < 0.01). The results demonstrated that prenatal women with high levels of anti-EV-D68 could transfer EV-68 NtAbs to neonates.

**Table2:** Relationship of EV-D68 NtAbs in blood samples collected from prenatal women and their neonates

The titer of prenatal women	The titer of neonates			Total titer
	1:8–1:64	1:96–1:512	>1:512	
1:8–1:64	8(20%)	2(5%)	–	10(25%)
1:96–1:512	2(5%)	20(50%)	2(5%)	24(60%)
>1:512	–	2(5%)	4(10%)	6(15%)
Total	10(25%)	24(60%)	6(15%)	40(100%)

### Seroprevalence rate and GMTs of EV-D68 NtAb in 0-year-old to 15-year-old children

**Fig. 3:**
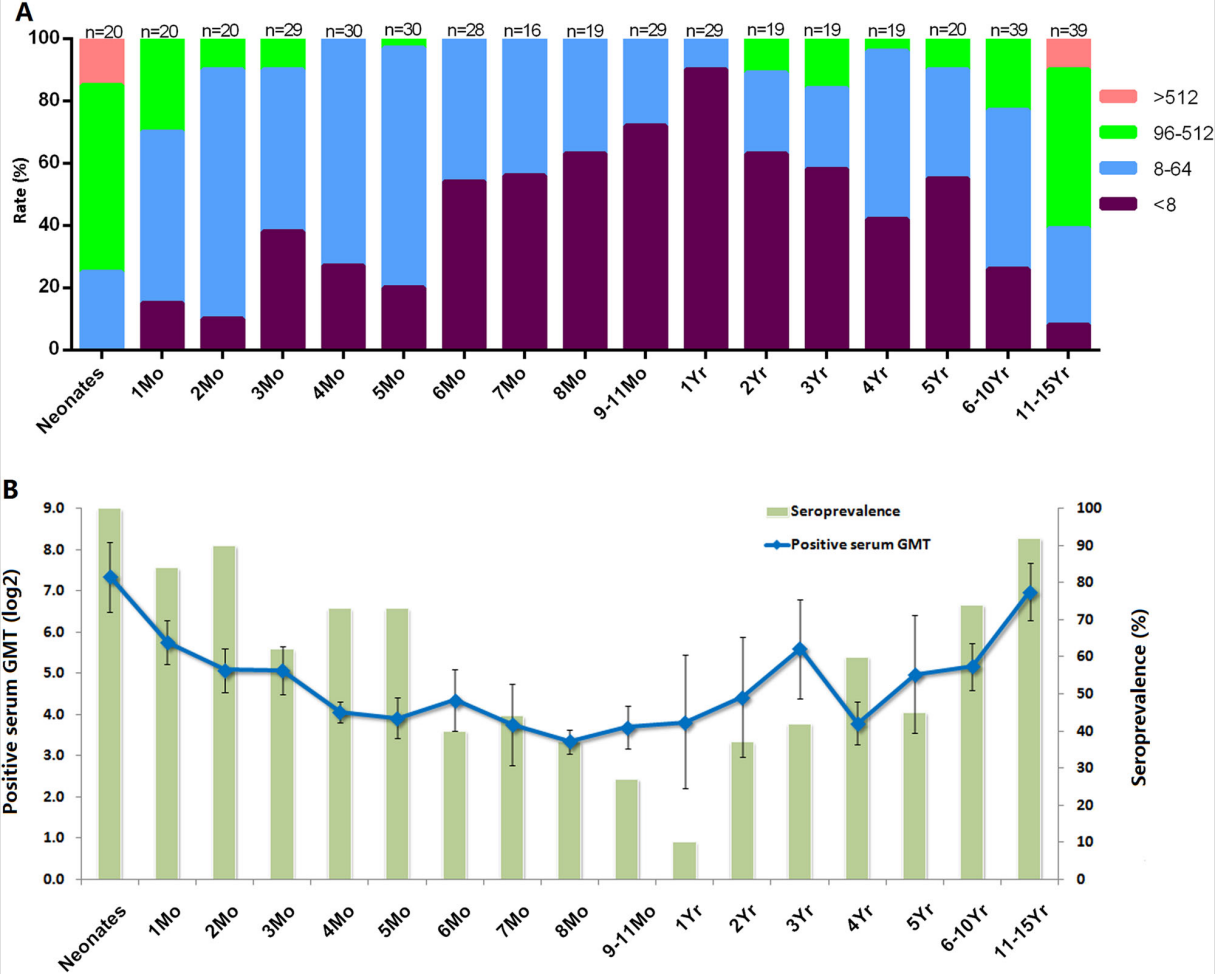
Seroprevalence rate and GMTs of anti-EV-D68 in blood samples of 0-year-old to 15-year-old children. **a** EV-D68 NtAb prevalence (100%) by age group; “n” was the number of the serum samples. **b** The GMT (positive serum) and seroprevalence rates were evaluated in different age groups

To understand the EV-D68 NtAbs distribution among newborns and juveniles, 16 cross-sectional serum samples from children ranging in age from 1 month to 11 to 15 years old were collected and detected against the synthetic viruses. Figure 3a shows that the titers were negatively correlated with age among neonates younger than 1 year old. The titers were positively correlated with age among children 1 to 15 years old, reaching a peak for 11-year-old to 15-year-old children, although that value remained lower than that of the neonates. The NtAbs titer was below 1:64 for all infants 6 months to 1 year old, making those infants susceptible to EV-D68 infection. For the 11-year-old to 15-year-old children, the NtAbs titers were even higher than 1:512, which may be caused by super-infection in this age group.

To further understand the NtAbs against EV-D68 in those newborns and children, the seroprevalence rates and GMTs (positive serum) were calculated (Fig. 3a, b). The seroprevalence rates of EV-D68 (NtAb ≥ 1:8) gradually decreased from 100% for newborns to 10% for infants younger than 1 year old (Fig. 3a). The seroprevalence rates gradually increased from 10% for children 1 year old to 92% for children 11 to 15 years old (Fig. 3a). Similar to the seroprevalence rates, the GMTs also followed the same trend (Fig. 3b), with 8-month-old infants having the lowest GMT. The GMTs decreased gradually from 162.3 for newborns (95 CI: 89.9–293.1) to 10.1 for 8-month-old infants (95 CI: 8.3–12.3). Then, the GMTs increased gradually from 10.1 for 8-month-old infants (95 CI: 8.3–12.3) to 126.0 for 11-year-old to 15-year-old children (95 CI: 78.0–203.6). Since the NtAb trend was slightly different for 8-month-old to 12-month-old infants, EV-D68 infection might occur in infants older than 8 months.

The overall seroprevalence rate of anti-EV-D68 antibodies was 59% (228/385, Table 3). For the four age groups in this study (1 to 5 months old, 6 months to 1 year old, 2 to 5 years old, and 6 to 11 years old), the seroprevalence rates were 79% (96/121), 20% (33/109), 44% (34/77), and 83% (65/78), respectively, whereas the GMTs were 25.2 (21.0–30.2), 15.0 (12.0–19.0), 24.7(17.15–35.6), and 71.9(50.8–101.8), respectively. The seroprevalence rates and GMTs of anti-EV-D68 antibodies were lower in the 6-month-old to 12-month-old infants than in the other age groups. The seroprevalence rates and GMTs of anti-EV-D68 antibodies in the 6-year-old to 15-year-old children were higher than those in 1-year-old to 5-year-old children.

**Table3:** Seroprevalence rates and GMTs of EV-D68 neutralizing antibodies among age groups

	1mo–5mo	6mo–1yr	2yr–5yr	6yr–15yr
GMT	25.2(21.0–30.2)^a^	15.0 (12.0–19.0)	24.7(17.15–35.6)^b^	71.9(50.8–101.8)^c^
Seroprevalence rates (%)	79(96/121)	20(33/109)	44 (34/77)	83(65/78)

^a^The seropositive rate of anti-EV-D68 in 6-month-old to 12-month-old infants was obviously lower than that in 1-month-old to 5-month-old infants, *p* = 0.0084

^b^The seropositive rate of anti-EV-D68 in 6-month-old to 12-month-old infants was obviously lower than that in 2-year-old to 5-year-old children, *p* = 0.0097

^c^The seropositive rate of anti-EV-D68 in 6-month-old to 12-month-old infants was obviously lower than that in 6-year-old to 15-year-old children, *p* = 0.0038

## Discussion

As the pathogen of HEV-D species, EV-D68 causes serious infectious diseases worldwide. Amino acid sequence sites on the surface of the viral capsid may explain differences of NtAb assays using different strains^20^.To analyze EV-D68 NtAbs for mothers, infants and children in China, the epidemic strain of China (KP240936, Beijing-R0132) was developed via reverse genetics, and the prototype strain (AY426531, Fermon) was purchased from ATCC. Our research showed that adult serum had higher NtAb GMTs against the synthetic strain than against the Fermon strain (*p* = 0.0378), although there was a high correlation between the two strains (*r* = 0.444,). Therefore, the synthetic strain was chosen as the challenge strain for the NtAb assay in this study.

In addition, a 100% seroprevalence rate was found for the Chinese population in 2015, similar to that in Finland in 2002 and in Beijing of mainland China in 2004 and in 2009. The GMTs were 166.3, 40–98, and 44.5 in China in 2015, Finland in 2002, and Beijing in 2004/2009, respectively^18, 21^. The results demonstrated that adults were widely infected with EV-D68 in both China and Finland. The difference in GMTs may be caused by several factors, including epidemic virus, strength, region and even the detection method of the virus strain. Therefore, the standard study of the EV-D68 NtAb assay must be performed to confirm the comparability of results from different regions at different times.

Maternal antibodies are the major immune mechanism that protects neonates against pathogens, but they can also interfere with the effects of immunization^22, 23^. The weakened rate in neonates was different in different regions because of the different levels of infection rates and titers. For example, based on epidemiology and the weakened rates of maternal antibodies, the World Health Organization (WHO) recommends that 2, 9, and 12 months are the appropriate prime time-points for immunizations of polio, measles, and hepatitis A virus vaccines, respectively. For HEV-A species, the maternal anti-EV71 and anti-CVA16 antibodies were evaluated^19^, and the EV71 vaccine was approved in China in 2015 (https://clinicaltrials.gov/). However, there have been no studies on mothers and their neonates regarding anti-EV-D68 antibodies. In our study, we described the maternally derived EV-D68 NtAbs in mothers and neonates. The GMT results demonstrated that newborn babies could obtain a high proportion and titer of EV-D68 NtAbs from their mothers. The situation was different for EV71 and CVA16. The GMTs of anti-EV-D68, anti-EV71, and anti-CVA16 were 162.3, 20.0, and 4.6 in neonates, respectively^19^, which might be due to the different sample sizes and ability to induce antibodies to these viruses.

As the neonates grew (from 0 to 8 months), the NtAbs seroprevalence and GMT decreased gradually. For the 8-month-old to 12-month-old infants, the GMT increased slowly while the seroprevalence decreased further. This inverse pattern of GMT and seroprevalence was caused by the decrease of maternally derived antibodies being partially offset by the acquired antibodies from infection. Our research showed that early infection might begin at 8 months in infants. The same pattern was also reported in another EV-D68 NtAb study for 6-month-old to 35-month-old infants, who were previously enrolled in a clinical trial to assess the immunogenicity of an enterovirus 71 (EV-A71) vaccine in Jiangsu Province (clinical trial No. NCT01508247) and who were included in a two-year follow-up study (January 2012–January 2014) in our laboratory. The lowest GMT was also found among 4-month-old to 8-month-old infants^24, 25^.

The titer distribution of these infants showed that the lowest NtAb titers were 80% negative and 100% lower than 1:64 in 6-month-old to 12-month-old infants. For children 1 year old and older, the NtAb indexes increased as the children grew older. Therefore, infants 6 months old and older should be treated as a susceptible population. Because of the declining protection from maternally derived antibodies, the risk of infection increases at this age with increasing exposure to crowds. A study in mainland China also showed that the infection primarily occurred among pre-school and school-aged children. In that study, GMTs were higher in the group aged 6.1–15 years old than in the group aged 0.5–6 years^18^. Our study had similar results. Most 2-year-old to 5-year-old children received pre-school education in kindergarten and showed a high seroprevalence rate of 44% and GMTs of 24.7. This finding was further supported by the high incidence of EV-D68 in pre-school children who were younger than 5 years. Hence, strengthening health improvement measures in kindergartens is necessary to effectively prevent infection. As the children grew, the seroprevalence rate increased to 83% and the GMT increased to 71.9 for 6-year-old to 15-year-old children (school-aged children). Such data demonstrate that immunity against EV-D68 was relatively high in children at this age, suggesting that EV-D68 infection was highly prevalent in this age group. The above results show that EV-D68 infection was age-dependent and that there might be multiple infections during the period of 6-year-old to 15-year-old.

In summary, this study demonstrated that a high level of maternal antibodies against EV-D68 was common in infants in China. From neonates to juveniles, the maternal antibodies decreased, but acquired antibodies from infection increased gradually. The lowest NtAb level of EV-D68 was found in 6-month-old to 12-month-old infants. Therefore, an early infection might occur in infants older than 8 months. A prime immunization course for vaccination must be established that can help protect this susceptible population. Because research on EV-D68 is limited in mainland China, surveillance on EV-D68 must be strengthened, and further study on EV-D68 virological characteristics, diagnostic method and vaccine development are necessary.

## Methods and materials

### Cells and virus

The EV-D68 prototype strain, Fermon (GenBank accession no.AY426531), was purchased from ATCC. The complete genome (GenBank accession no.KP240936, Beijing-R0132) was constructed under the pBluescriptII SK-vector (Tai he gene, Beijing, China). 5′UTR and 3′UTR were added to the T7 promoter of the ribozyme and the T7 terminator, respectively. The RNA polymerase gene sequence was constructed under the CMV promoter (pcDNA6.1 vector). The virus was grown in the monolayer Rhuman Embryonic Kidney (293T cells) and loaded in a 6-well plate (Corning) with Dulbecco’s modified Eagle medium (DMEM) and 10% fetal bovine serum (FBS). Lipo 2000 reagent (Invitrogen) was used to co-transfect 293T cells with a plasmid of the synthetic genome and the RNA polymerase gene according to the manufacturer’s procedure. The resulting cells were cultured for 3–4 days before the cell suspensions were harvested. The collected cells were frozen and thawed 3 times, and the genomes were collected with centrifugation at 12,000 rpm for 10 min. The resulting viral genomes were mixed with a monolayer of human rhabdomyoma cells (RD cells) with DMEM in 2% FBS. The synthetic virus was harvested, and the tissue culture infective dose of 50% (TCID_50_) was determined.

### Human subjects and serum samples

Serum specimens were collected from prenatal women and their neonates at birth to determine the titer of NtAbs against EV-D68. A total of 20 pairs of serum samples were collected. To further investigate the age-related seroprevalence of NtAbs against EV-D68 in infants and young children, serum specimens were also collected from different age groups: 1, 2, 3, 4, 5, 6,7, 8, and 9 to 11 months, and 1, 2, 3, 4, 5, 6 to 10, and 11 to 15 years, respectively. All 405 serum samples were collected randomly in Donghai County in August 2010.

To evaluate the seroprevalence of EV-D68 infection among adults, 50 adult serum samples were collected in Henan province, China, in 2015. Informed consent was obtained from either the participants or their guardians. This study was approved by the ethical review committee of the National Institutes for Food and Drug Control. The serum was separated immediately after collection and stored at −80 °C.

### Measurement of neutralizing antibodies

In our previous study, we had established Standard Operating Procedures (SOPs) for the determination of enterovirus A71 and coxsackievirus A16 NtAbs^26, 27^. EV-D68 NtAbs were detected in this study based on this previously reported method. Briefly, blood samples were diluted to a ratio of 1:8 and then inactivated at 56 ℃ for 30 min. The inactivated serum was serially diluted from 1:8 to 1:1024 and placed in a 96-microtiter plate. After 100 TCID_50_ of EV-D68 was added to the above plate, the mixture was incubated for 2 h at 33 °C in a CO_2_ incubator to allow the antibodies to bind to the virus. After the incubation, the RD cell suspension (2–3 × 10^5^cells/ml) was added. The cell control, serum control, virus control and virus back titration (if the result of the back drop was 32~320 TCID_50_/well, the test was considered a success) were included in each plate, and the plate was placed in a CO_2_ incubator at 33 °C for 7 days. Cytopathogenicity was observed using microscopy to identify the titers of NtAbs, defined as the inhibition of 50% CPE.

### Statistical analysis

The titers of the neutralizing antibodies were log-transformed to calculate the GMTs with 95% confidence intervals. All statistical analyses were performed using the GraphPad Prism software package. Group comparisons were performed using the Student’s *t* test, and a *p* value < 0.05 was considered statistically significant. The titers above 1:1024 were designated as 1:1024.
